# Programming co-assembled peptide nanofiber morphology via anionic amino acid type: Insights from molecular dynamics simulations

**DOI:** 10.1371/journal.pcbi.1011685

**Published:** 2023-12-04

**Authors:** Xin Y. Dong, Renjie Liu, Dillon T. Seroski, Gregory A. Hudalla, Carol K. Hall

**Affiliations:** 1 Department of Chemical and Biomolecular Engineering, North Carolina State University, Raleigh, North Carolina, United States of America; 2 Department of Biomedical Engineering, University of Florida, Gainesville, Florida, United States of America; Weizmann Institute of Science, ISRAEL

## Abstract

Co-assembling peptides can be crafted into supramolecular biomaterials for use in biotechnological applications, such as cell culture scaffolds, drug delivery, biosensors, and tissue engineering. Peptide co-assembly refers to the spontaneous organization of two different peptides into a supramolecular architecture. Here we use molecular dynamics simulations to quantify the effect of anionic amino acid type on co-assembly dynamics and nanofiber structure in binary CATCH(+/-) peptide systems. CATCH peptide sequences follow a general pattern: CQCFCFCFCQC, where all C’s are either a positively charged or a negatively charged amino acid. Specifically, we investigate the effect of substituting aspartic acid residues for the glutamic acid residues in the established CATCH(6E-) molecule, while keeping CATCH(6K+) unchanged. Our results show that structures consisting of CATCH(6K+) and CATCH(6D-) form flatter β-sheets, have stronger interactions between charged residues on opposing β-sheet faces, and have slower co-assembly kinetics than structures consisting of CATCH(6K+) and CATCH(6E-). Knowledge of the effect of sidechain type on assembly dynamics and fibrillar structure can help guide the development of advanced biomaterials and grant insight into sequence-to-structure relationships.

## Author summary

Peptides are great building blocks for supramolecular biomaterials—with applications in drug delivery, tissue engineering, and so forth. Peptide co-assembly refers to the spontaneous coming together of mixtures containing two different peptides to form organized structures. CATCH (Co-Assembly Tags based on CHarge complementarity) are mixtures of oppositely charged peptides that co-assemble into β-sheet nanofibers. CATCH peptide sequences follow a general pattern: CQCFCFCFCQC, where all C’s are either a positively charged or a negatively charged amino acid. In this study, we use molecular dynamics simulations to determine the effect of negative amino acid type on co-assembly dynamics and nanofiber structure, specifically the difference between incorporating glutamic acid (E-) and aspartic acid (D-). Our results show that structures created using aspartic acid (D-) form flatter β-sheets and have slower co-assembly kinetics than structures created with glutamic acid (E-). Understanding the effect of different amino acid types on peptide assembly and nanofiber properties will allow us to design biomaterials with predetermined structural properties.

## Introduction

Peptides have been extensively used as building blocks for supramolecular biomaterials in applications ranging from drug delivery and tissue engineering, to biosensors [1,2]. Peptide-based hydrogels are appealing due to their biocompatibility, biodegradability, and low toxicity. Peptide-based biomaterials can be formed from a single component (“self-assembly”) or through a blend of components (“co-assembly”). Here we describe a system that can be made via selective co-assembly, which occurs when peptides A and B co-assemble in solution, but remain in a random-coil state when separated. Selective co-assembly allows for control of the assembly pathway and, in turn, enables precise formation of nanofiber structures, resulting in a hydrogel with predictable and uniform properties.

CATCH (Co-Assembly Tags based on CHarge complementarity) are binary systems of oppositely charged synthetic peptides that selectively co-assemble into β-sheet nanofibers [3]. Charge complementarity drives CATCH peptide co-assembly; attraction between oppositely-charged peptides promotes cooperative co-assembly, while repulsion between like-charged peptides discourages self-assembly. CATCH peptides are cationic and anionic variants of Q11[QQKFQFQFEQQ]. The alternating motif of hydrophobic and hydrophilic residues in Q11 is a common feature in self-assembling peptides and is preserved in the CATCH peptides [4] The original pair of CATCH peptides reported were: CATCH(4+), (Ac-QQKFKFKFKQQ-Am) and CATCH(6−), (Ac-EQEFEFEFEQE-Am), where the number and sign denote the overall charge of the peptide as measured by the number of (positively-charged) lysine (K) or (negatively-charged) glutamic acid (E) residues [4]. CATCH peptides have been used successfully to immobilize functional proteins within macroscopic hydrogels [4]. The effect of the total charge on the co-assembly of pairs of CATCH peptides was determined by Seroski et. al who investigated CATCH(2+/2-), (4+/4-), and (6+/6-) peptide systems. They found that increasing the number of charged residues within each peptide results in an increased rate of co-assembly [5]. However, these studies did not explore the effects of replacing the type of cationic or anionic residues within CATCH peptides on their co-assembly.

**Fig 1:**
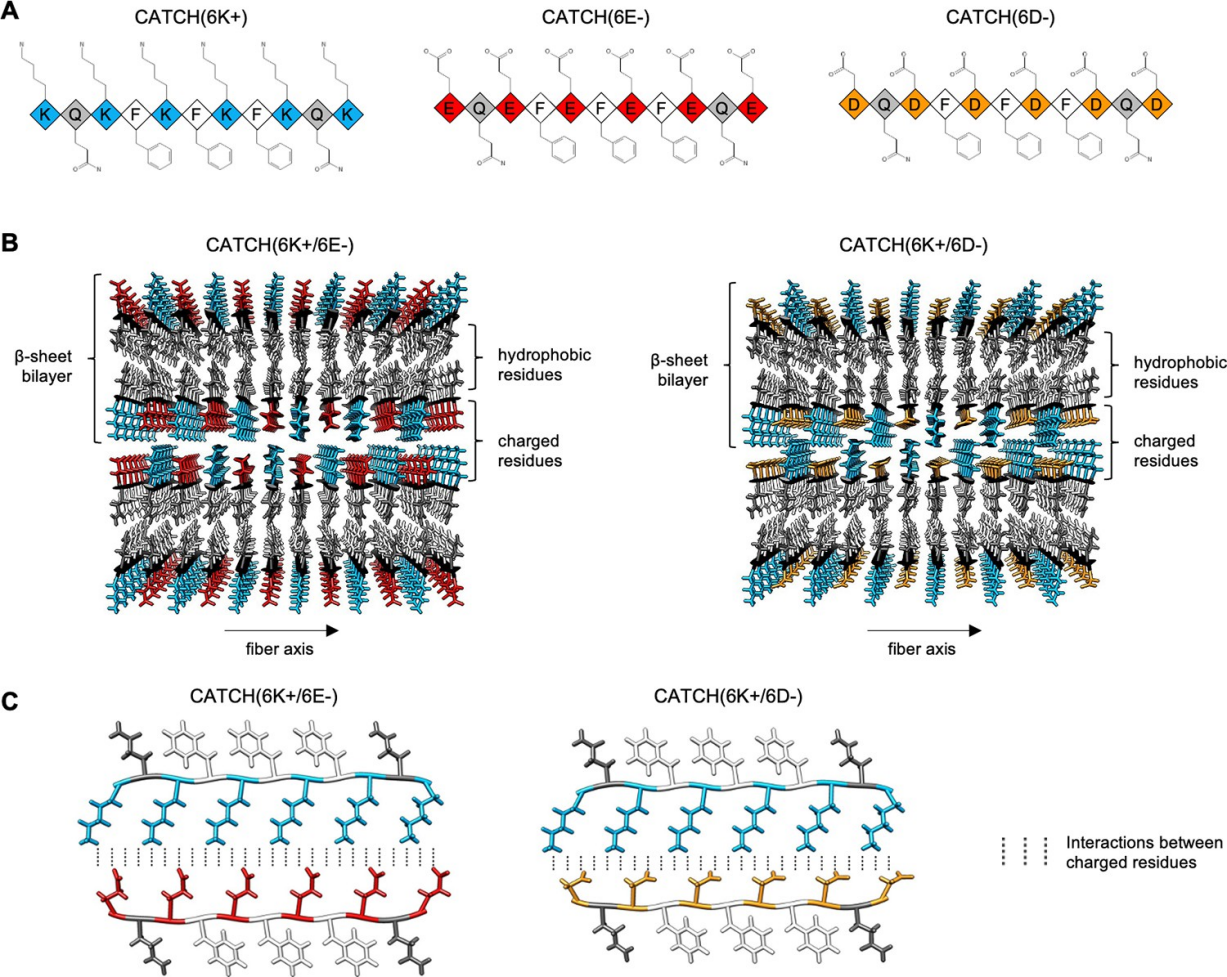
**(A)** Schematic of CATCH peptide sequence and sidechain structure for CATCH(6K+) in blue, (6E-) in red and (6D-) in orange, **(B)** Front view of CATCH(6K+/6E-) and CATCH(6K+/6D-) fibril showing two stacked bilayer starting structures built in PACKMOL and rendered in Chimera.[10,11] Sidechain structures are represented using sticks and colored based on the schematic from (A). Backbones are represented using black arrows and are directed into or out of the page. **(C)** Side view of CATCH(6K+/6E-) and CATCH(6K+/6D-) system.

**Fig 2:**
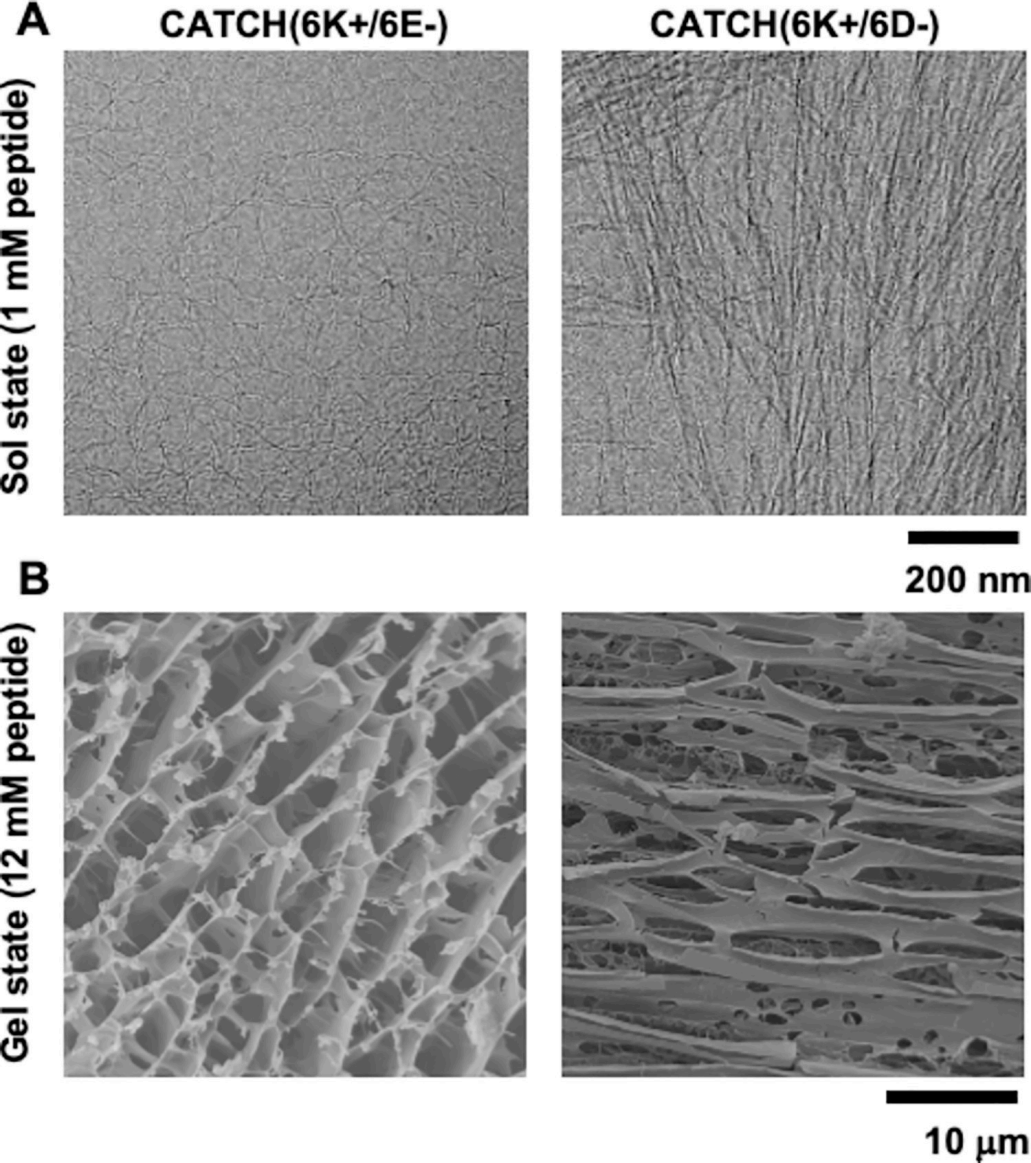
Morphology of CATCH(6K+/6E-) and CATCH(6K+/6D-) co-assemblies. (A) Cryogenic TEM micrographs of CATCH(6K+/6E-) and CATCH(6K+/6D-) in the sol state (1 mM total peptide). (B) Cryogenic SEM micrographs of CATCH(6K+/6E-) and CATCH(6K+/6D-) in the gel state (12 mM total peptide).

Here we study the effect of sidechain type on CATCH co-assembly [4]. We substitute negatively-charged aspartic acid residues (D) for the negatively-charged glutamic acid residues (E) in CATCH(6+/6-) pairs. Aspartic acid (D) is a convenient substitute for glutamic acid (E), as it is one methylene group shorter (Fig 1A). The positively-charged amino acid residue, lysine, remains the same. We will refer to the CATCH(6+/6-) mixture with glutamic acid as CATCH(6K+/6E-) (KQKFKFKFKQK/EQEFEFEFEQE), and the mixture with aspartic acid residues as CATCH(6K+/6D-) (KQKFKFKFKQK/DQDFDFDFDQD). Experiments performed by Liu et. al show that CATCH(6K+/6E-) and CATCH(6K+/6D-) form hydrogels with different structural and mechanical properties. Cryogenic scanning electron microscopy and conventional transmission electron microscopy (TEM) (Fig 2) show that CATCH(6K+/6E-) nanofibers are randomly entangled, whereas CATCH(6K+/6D-) form multi-layer stacks of aligned fibrils [6]. We hypothesize that the mismatched lengths of the charged residues in CATCH(6K+/6D-) create an incentive for two bilayers to stack together (Fig 1B and 1C). Thioflavin T analyses show that CATCH(6K+/6E-) assembles at a faster rate than CATCH(6K+/6D-), suggesting a difference in interaction strength between the (K+/E-) pair and the (K+/D-) pair.

The aim of this work is to determine the effect of sidechain type on peptide co-assembly and how this corresponds to the nanofiber structures and morphologies observed in experiments. A computational approach is taken. Atomistic molecular dynamics simulation is used to analyze sidechain-sidechain interactions in detail as this affords a closer look (higher resolution) than can be obtained in biophysical experiments or in coarse-grained simulations. Atomistic simulations of a single bilayer and of two stacked bilayers (two bilayers stacked upon one another) are performed and analyzed for each CATCH(6K+/6E+) and CATCH(6K+/6D-) system. Single bilayers are also simulated to predict the fibril structure for each peptide pair. The two stacked bilayer simulations are used to quantify the sidechain-sidechain interactions between charged residues that sit between the bilayers. We also perform simulations of single monomeric peptides to determine the native monomeric state for CATCH(6K+), (6D-), and (6E-). A coarse-grained simulations approach, discontinuous molecular dynamics (DMD) with the PRIME20 forcefield, is used to investigate assembly kinetics and pathway for large CATCH(6K+/6E-) systems and CATCH(6K+/6D-) systems starting from random-coil conformations. DMD/PRIME20 simulations allow access to timescales that are not available through traditional atomistic molecular dynamics, and spatial resolution that is not accessible through biophysical measurements.

Highlights of our results include the following: Single bilayer atomistic simulations show that CATCH(6K+/6E-) has a more pronounced twist than CATCH(6K+/6D-), providing a possible explanation for the experimentally-observed differences in nanofiber thickness between the two. Atomistic simulations of the two stacked bilayers show weaker van der Waals and electrostatic interactions between charged residues (on the second and third layer) for CATCH(6K+/6E-) than for CATCH(6K+/6D-). Atomistic simulations of the two separated bilayers show fewer number of contacts between charged residues (on the second and third layer) for CATCH(6K+/6E-) than for CATCH(6K+/6D-). Analysis of DMD/PRIME20 results show that CATCH(6K+/6E-) monomers co-assemble at a faster rate than CATCH(6K+/6D-) monomers, in agreement with experimental thioflavin T analyses. Discordant helical segments found in the CATCH(6E-) single peptide atomistic REMD simulation offer an additional possible explanation for the fast co-assembly observed for CATCH(6K+/6E-). Visualization of CATCH(6K+/6D-) DMD results shows that some β-barrel intermediates undergo a β-barrel-to-β-sheet conformation change during β-sheet assembly. Overall, our results suggest that the anionic sidechain composition in CATCH(6K+/6E-) results in random entanglement of nanofibers, while the anionic sidechain composition in CATCH(6K+/6D-) results in multi-layer stacks of aligned fibrils.

## Methods

### Explicit-solvent atomistic molecular dynamics simulation

Explicit-solvent atomistic MD simulations at T = 310 K are carried out in the canonical ensemble using the AMBER package with the AMBER ff14SB force field [7] to quantify the sidechain-sidechain interactions between CATCH peptide pairs for CATCH(6K+/6E-) and for CATCH(6K+/6D-). Temperature is maintained using the Langevin thermostat [8]. The SHAKE algorithm is used to maintain bond length constraints on bonds involving hydrogens [9].

**Fig 3:**
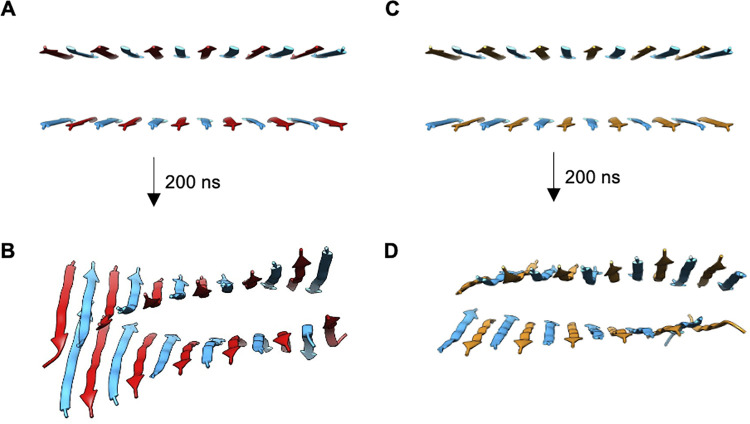
Snapshots of **(A-B)** CATCH(6K+/6E-) and **(C-D)** CATCH(6K+/6D-) bilayers before and after 200 ns of simulation. Final structure of CATCH(6K+/6E-) and CATCH(6K+/6D-) have an average twist of -3.55 and -2.22° between neighboring peptides, respectively.

**Fig 4:**
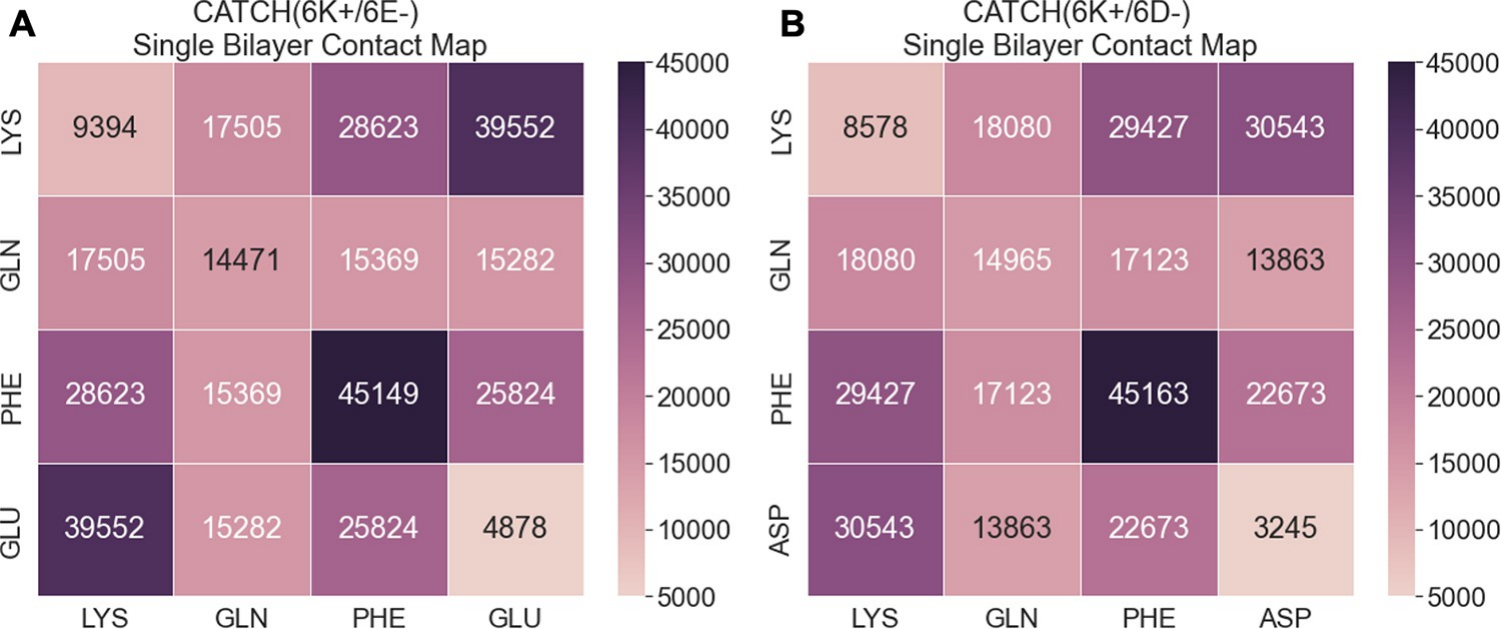
Contact map for (A) CATCH(6K+/6E-) and (B) CATCH(6K+/6D-). Contacts are counted for all atoms in each single bilayer system and grouped by residue. Contacts between two atoms were determined using a distance cutoff of 7Å. Values reported are averaged over three independent MD simulations.

Four different atomistic simulation configurations were built: (1) two stacked bilayers—two bilayers stacked upon one another, (2) two separated bilayers—two bilayers separated by a distance of ~13Å measured from the surface of each bilayer, (3) a single bilayer, and (4) a single peptide. The AMBER tLEaP program was used to build the peptide sequence; the N-terminal was capped with an acetyl group and the C-terminal was capped with a methyl group. Phi-psi angles were modified to conform to an antiparallel β-strand using Chimera [10]. PACKMOL was used to arrange peptides to create a single bilayer, the two stacked bilayers, and the two separated bilayers (Figs 3 and 4) [11]. The single bilayer was built with 12 peptides in each in-register antiparallel β-sheet. The two stacked bilayers and the two separated bilayers models consist of four in-register antiparallel β-sheet layers, with 12 peptides in each layer stacked on top of one another. In all bilayer systems, the neighboring β-strands were spaced ~5 Å apart and the β-sheets within a bilayer were spaced ~13Å apart (to promote hydrophobic interactions). The inter-strand spacing and antiparallel orientation of our model is validated by previous PITHIRDS-CT and FTIR work [12]. Each CATCH bilayer structure was solvated in a periodic truncated octahedral box containing TIP3P water with a 12 Å buffer [13]. Single peptide simulations were started in an extended conformation with phi-psi angles of -180 and 180° respectively.

Three independent simulations were run for the single bilayer systems, the two stacked bilayers, and the two separated bilayers. Each system was subjected to thermal annealing steps prior to the production run. The protocol for our atomistic MD simulations was as follows: (1) a 1000-step energy minimization using the steepest descent method was performed on the solvent molecules with the peptide structure constrained by a force of 500 kcal/mol. (2) A 2500-step energy minimization was performed on all atoms in the system. (3) Systems were brought up to 310 K through a series of heating stages over the course of 50 ps. (4) Thermal annealing was performed in the following steps: heat from 310 K to 400 K, equilibrate at 400 K, heat from 400 K to 500 K, equilibrate at 500 K, cool from 500 K to 310 K. Each step was performed for 100 ps. (5) 200 ns production runs were conducted in the NPT ensemble at 310 K. Root-mean-squared deviation (RMSD) analysis was performed on the last 10 ns of each simulation trajectory using the output structure from the minimization step to verify that the trajectory had reached an equilibrated state.

All-atom implicit-solvent temperature REMD simulations for a single CATCH(6K+), (6E-), and (6D-) peptide were carried out using the AMBER package. Peptides were parameterized with the ff14SB forcefield [7]. For an exchange probability of ~0.25, with temperatures ranging from 310 to 600 K, eight replicas were generated for each CATCH peptide [14]. Chirality restraints were generated for each system to avoid a chirality inversion. Langevin dynamics was employed for temperature control [8]. Constraints were maintained using the SHAKE algorithm [9]. Prior to simulation, each system underwent energy minimization using the steepest descent algorithm for 500 cycles. Each system was then equilibrated at the desired temperature for 200 ps. Exchange attempts were made every 2 ps between adjacent replicas. Exchanges are accepted or rejected based on a Metropolis acceptance criterion that satisfies the detailed balance. Each simulation had a total of 100,000 exchange attempts for a total simulation time of 200 ns. Secondary structure content for REMD simulation results were calculated using the DSSP algorithm [15,16].

The implicit-solvent molecular mechanics/Generalized Born Surface Area (MM/GBSA) [17] approach was used to analyze the last 5 ns of the simulation trajectories to calculate the interaction energy between charged residues. MMGBSA is typically used in drug design to determine the binding affinity between a ligand and receptor by calculating the binding free energy (ΔG_binding_). For the MMGBSA analysis, we define the top bilayer to be the “ligand,” the bottom bilayer to be the “receptor,” and the overall structure to be the “complex." Here we neglect entropy and focus on the interaction energy, as our system is quite large compared to the small molecules typically modeled. We calculated the van der Waals and electrostatic interaction energies between charged residues on the second and third β-sheets that result when the two bilayers stack together and the exposed charged residues between the bilayers interact. In this case, VDW and ELE are defined to be the difference in the van der Waals and electrostatic energies, respectively, between two bilayers before and after they stack together.

The linear interaction energy (LIE) approach [18] was used to calculate the VDW interaction energies between the sidechains of intra-sheet and inter-sheet neighboring charged residues for the last 5 ns of the simulation trajectories. Similar to MMGBSA, it is typically used to predict the binding affinity of protein-ligand complexes. We define one group of residues to be the “ligand” and the other group to be the “receptor.” Here we utilize LIE to calculate the VDW interactions between two specific groups of sidechain atoms.

### Coarse-grained DMD simulations

Implicit-solvent discontinuous molecular dynamics (DMD) simulations are carried out using the PRIME20 forcefield—designed specifically for modeling peptide aggregation. In PRIME20, amino acids are modeled by four spheres: three backbone spheres NH, Cα, and CO and one sidechain sphere R. Each sidechain sphere on each amino acid has a distinct size (effective van der Waals radius) and a distinct geometric structure (R-NH, R-Cα, and R-CO bond lengths). The two major non-bonded interactions in PRIME20 are directional hydrogen bonding interactions between backbone NH and CO spheres, and (non-directional) interactions between two sidechain R spheres. Both are modeled as square-well interactions. Polar, charge–charge, and hydrophobic interactions between amino acid sidechains are described using a combination of 210 different square-well widths and 19 different square-well depths [19]. All other interactions are modeled using a hard-sphere potential. Hard-sphere diameters, square-well widths, and square-well depths were determined by Cheon et. al using a perceptron learning algorithm [19]. A detailed description of the geometric and energetic parameters of the PRIME20 model is provided in earlier work [19–21].

Three independent simulations of CATCH(6K+/6E-) and CATCH(6K+/6D-) were run. Each system contained a total of 200 peptides—100 positively-charged peptides and 100 negatively-charged peptides—randomly distributed in a cubic box with a side length of 321 Å for a peptide concentration of 20 mM. All simulations were carried out for approximately 16 μs in the canonical ensemble. The Andersen thermostat was employed to maintain the simulation at a constant reduced temperature T* of 0.18, roughly corresponding to 296 K [22,23]. The reduced temperature in our system is defined as T* = k_B_T/ε_HB_, where ε_HB_ = 12.47 kJ/mol is the hydrogen bonding energy [22].

Elements of graph theory are used to determine the rate of oligomerization and fibril formation [12]. Each oligomer cluster is defined as a network of peptides that are connected through a combination of hydrophobic and/or hydrogen bonding interactions. The fibril is considered to be the final β-sheet formed at the end of the simulation. A pair of peptides is considered “connected” if one of two conditions is met: (1) there are at least five hydrogen bonds between the pair of peptides, or (2) there are at least two hydrophobic interactions. For our system of CATCH peptides, these conditions are considered sufficient to accurately track the formation of oligomers and fibril formation over the course of a simulation.

Data generated from DMD and MD simulations are provided on Dryad [24].

## Dryad DOI

10.5061/dryad.5mkkwh7bp.

## Results and discussion

### Analysis of single CATCH bilayer geometry and intra-sheet sidechain-sidechain interactions

Three independent explicit-solvent atomistic MD simulations for a single CATCH(6K+/6E-) bilayer structure and for a single CATCH(6K+/6D-) bilayer structure were carried out to determine the bilayer geometry and intra-sheet sidechain-sidechain interactions (Fig 3A–3D). The initial configuration for each CATCH mixture was an “ideal” structure with 12 in-register antiparallel peptides in each layer. Over the course of the 200 ns simulation, the sidechains in each structure relaxed into a more realistic geometry.

The longer anionic sidechain in CATCH(6K+/6E-) leads to a more pronounced left-handed bilayer twist in CATCH(6K+/6E-) than in the CATCH(6K+/6D-) bilayer (Fig 3B and 3D). Here, we define the twist in terms of the angle between two neighboring β-strands. This angle was measured by calculating the line of best fit for the set of Cα atoms in each peptide, defining that line to be a vector with end points at the N- and the C-terminal Cα atoms, and then measuring the angle between the two resulting vectors. The CATCH(6K+/6E-) simulations resulted in a twisted bilayer, while the CATCH(6K+/6D-) simulations resulted in a relatively flat bilayer. The average angle of twist between the nearest neighbor peptides for CATCH(6K+/6E-) and CATCH(6K+/6D-) were -3.55°and -2.22°, respectively. The left-handed β-sheet twist (denoted by the negative sign) observed in the CATCH(6K+/6E-) β-sheet bilayer is inherent to antiparallel β-sheets. This twisting phenomenon can be attributed to the chirality of the amino acids, the inter-strand backbone hydrogen bonding, and inter-strand sidechain interactions [25–31].

Over the course of the last 5 ns of the MD simulation, CATCH(6K+/6E-) and CATCH(6K+/6D-) possess roughly the same amount of backbone hydrogen bonds and salt bridge interactions. Salt bridges are defined to be interactions between two oppositely charged groups containing at least two heavy atoms within hydrogen bonding distance of each other. For simplicity, we define salt bridges to be between any oxygen on the carboxylate group (in D or E) and any hydrogen on the ammonium groups (in K) that satisfy a distance cutoff of 3Å and an angle cutoff of 135°. Salt bridges in our simulations had an average length of ~2.8 Å and an average bonding angle of ~156°. Salt bridge interactions were calculated using the LIE approach (Table 1); VDW and ELE interactions were calculated between the atoms on lysine’s ammonium group and the atoms glutamic acid and aspartic acid’s carboxylic acid group. CATCH(6K+/6E-) had less favorable VDW interactions than (6K+/6D-), but more favorable ELE interactions; CATCH(6K+/6E-) and (6K+/6D-) had ELE interactions of -5402 and -4410 kcal/mol, respectively. However, when considering the charged residues as a whole (excluding the backbone atoms), CATCH(6K+/6E-) had more favorable VDW interactions than (6K+/6D-), -125.9 vs. -85.8 kcal/mol.

**Table 1:** Summary of hydrogen bonding and LIE analysis for CATCH single bilayer structures. Hydrogen bonds and salt bridges were calculated using geometric criteria: an angle cutoff of 135° and a distance cutoff of 3.0Å. Salt bridges were defined to be between the hydrogens on lysine’s ammonium group and the oxygens on glutamic acid or on aspartic acid’s α-carboxylic acid group. Salt bridge VDW and ELE interactions were calculated between the atoms on the lysine’s ammonium group and the atoms on glutamic acid and aspartic acid’s carboxylic acid group. VDW interactions between charged residues are calculated using the LIE approach and exclude backbone atoms. Values listed are averaged over three independent simulations.

System	No. of backbone hydrogen bonds	No. of salt bridges	Salt bridge VDW interactions (kcal/mol)	Salt bridge ELE interactions (kcal/mol)	VDW interactions between charged residues (kcal/mol)
CATCH(6K+/6E-)	128.2 ± 0.5	34.9 ± 1.8	54.3± 4.4	-5402.0± 100	-125.9 ± 3.8
CATCH(6K+/6D-)	119.2 ± 4.8	27.6 ± 1.3	42.3± 2.1	-4410.3± 140	-85.8 ± 1.8

The difference in β-sheet twisting for the CATCH(6K+/6E-) and CATCH(6K+/6D-) single bilayers may be explained by how each CATCH pair organizes its β-sheet structure to maximize hydrogen bonding and salt bridge interactions. In CATCH(6K+/6E-), the sidechains of lysine (K) and glutamic acid (E) are of similar length, facilitating a salt bridge interaction between the charged groups on the ends of the charged residues. In CATCH(6K+/6D-), the sidechains of lysine (K) and aspartic acid (D) are of mismatched length. Given that the salt bridge interactions in both CATCH systems have similar geometry in terms of bond length and bond angle, it is likely that CATCH(6K+/6D-) accommodates the mismatched sidechain lengths by hindering its backbone from forming an inherent left-handed twist. The relationship between sidechain length and β-sheet conformation observed in the CATCH system is consistent with a previous quantitative and experimental studies that have shown that β-strands containing glutamic acid have a greater propensity for twisting than those containing aspartic acid [32–34].

To further interrogate the interactions within the single bilayer structure, we calculated the number of contacts between residues within the structure and the overall VDW interactions between the cationic sidechains and the anionic sidechains. Contacts are defined to be any two atoms within 7Å of one another. The greatest number of contacts for both CATCH(6K+/6E-) and (6K+/6D-) was between the phenylalanine residues, 45,149 and 45,163 contacts, respectively (Fig 4). This is expected as the phenylalanine residues make up the hydrophobic core of the bilayer structures. The second greatest number of contacts in each system was between the cationic sidechains and the anionic sidechains. CATCH(6K+/6E-) had 39,552 contacts and a VDW interaction of -125.9 kcal/mol between the lysine and glutamic acid sidechains; (6K+/6D-) had 30,543 contacts and a VDW interaction of -85.8 kcal/mol between the lysine and aspartic acid sidechains. The additional methylene group in glutamic acid (E) in the CATCH(6K+/6E-) pair leads to more contacts and stronger inter-strand interactions between charged residues than in the CATCH(6K+/6D-) pair. The stronger interaction for the K/E sidechain pair compared to the K/D sidechain pair is consistent with pair correlations calculated by Wouters et al. and isothermal titration calorimetry experiments performed by Petrauskas et al. [35,36] The relationship between strong inter-strand interactions and the β-sheet twisting observed in CATCH(6K+/6E-) is consistent with previous experimental studies that suggest that increased interactions between intra-sheet neighboring sidechains is related to increased twisting [26–28].

### Evaluation of face-to-face interactions between the two stacked CATCH bilayers

**Fig 5:**
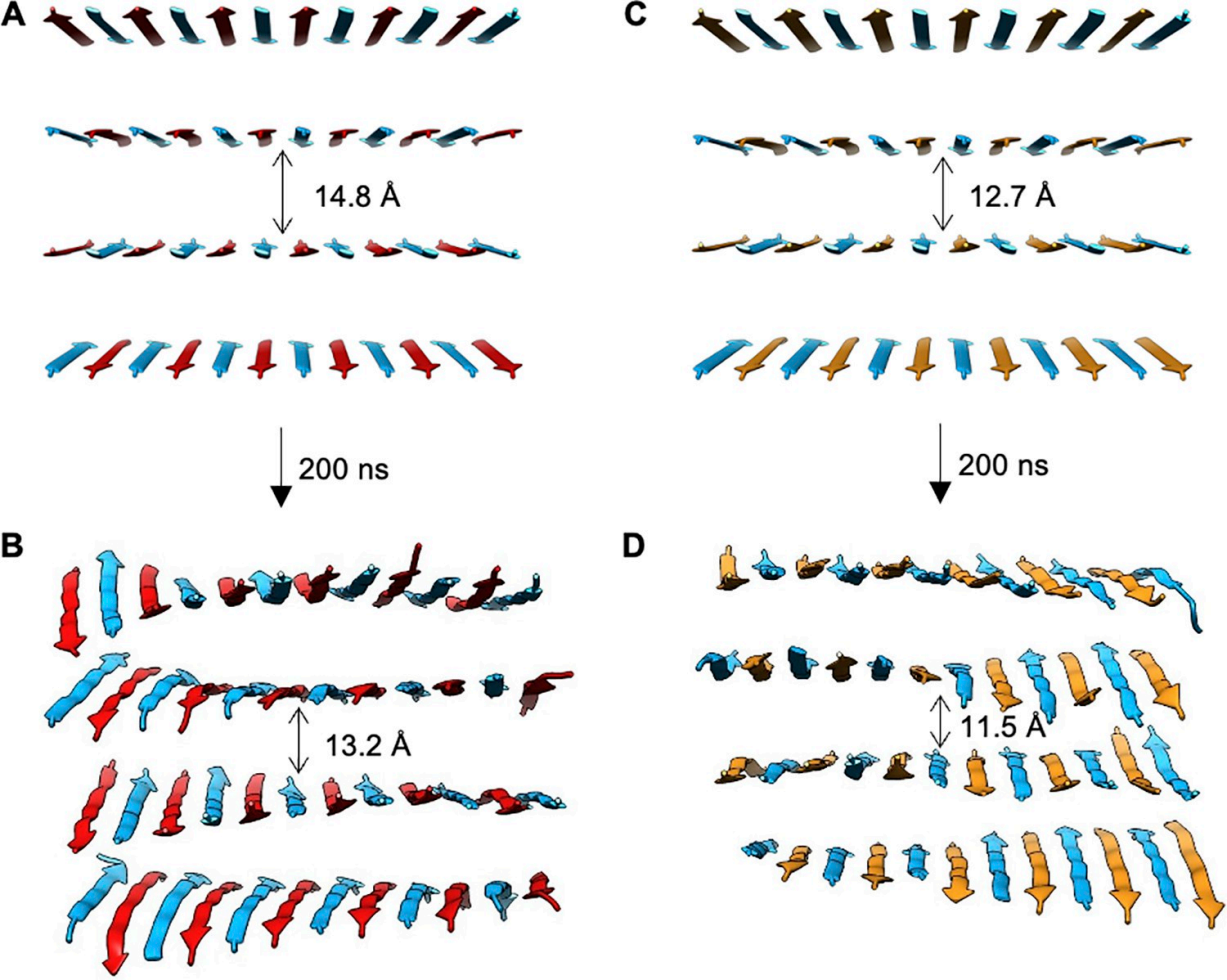
Snapshots of **(A-B)** CATCH(6K+/6E-) and **(C-D)** CATCH(6K+/6D-) two stacked bilayers before and after 200 ns of simulation. Distances between the second and third layer of each structure are indicated.

Three independent explicit-solvent atomistic MD simulations were carried out for a CATCH(6K+/6E-) mixture and for a CATCH(6K+/6D-) mixture, with each arranged in the two-stacked bilayer configuration. Each simulation started from a pre-formed “ideal” structure (Fig 5A–5D). Intra-sheet β-strands were spaced ~5 Å apart (measuring from the backbone center) to promote backbone hydrogen bonding between neighboring β-strands. The initial bilayers for both CATCH(6K+/6E-) and CATCH(6K+/6D-) were spaced ~3 Å apart, measuring between the end of the sidechains facing inward on the second and third β-sheet. As the simulation progressed, the van der Waals and electrostatic interactions between the charged residues drove the bilayers closer together and stabilized the structure, as expected.

Atomistic simulations of the two stacked bilayer systems showed tighter packing between the second and third layer for CATCH(6K+/6D-) than for CATCH(6K+/6E-). The distance between the second and third layer was measured by fitting the backbone atoms of each sheet to a plane and measuring the distance between the centroids of the two fitted planes. The average distance between the second and third β-sheet for CATCH(6K+/6E-) and for CATCH(6K+/6D-) changed over the course of the simulation from an initial distance of 14.8 and 12.7 Å to a final distance of 13.2 and 11.5 Å, respectively. Thus, the CATCH(6K+/6E-) bilayers were further apart than the CATCH(6K+/6E-) bilayers by roughly 2 Å. The longer anionic sidechain (E) in CATCH(6K+/6E-) acts as a physical barrier in the stacking process, while the shorter anionic sidechain (D) in CATCH(6K+/6D-) allows the charged residues to interdigitate in an alternating manner, bringing them closer together. In CATCH(6K+/6D-) the complementary charged residues between the second and third layer create a tight steric zipper that draws the sheets closer together [37,38].

Atomistic MD simulations of the two stacked bilayer CATCH systems show that CATCH(6K+/6D-) bilayers have more favorable face-to-face interactions than CATCH(6K+/6E-) bilayers (Table 2). MMGBSA analysis is used to calculate the van der Waals (VDW) and electrostatic (ELE) interaction energies between the two CATCH bilayers as a result of stacking (Table 2). Both the VDW and ELE interactions are more negative in CATCH(6K+/6D-) than in CATCH(6K+/6E-), suggesting that CATCH(6K+/6D-) has more favorable interactions between the two bilayers and a greater energy incentive for bilayer stacking than CATCH(6K+/6E-). LIE analysis is used to calculate the pairwise VDW interactions between the sidechains of the exposed charged residues (excluding the backbone atoms) on the second and third layer. CATCH(6K+/6D-) experiences a VDW interaction that is 2-fold stronger than for CATCH(6K+/6E-), -49.8 vs -20.7 kcal/mol, in agreement with the MMGBSA results (Table 2). The contrast in interaction energies between the two CATCH systems can be explained by the physical arrangement of the stacked bilayers. The two stacked bilayer simulations have shown that CATCH(6K+/6D-) can stack more closely together than CATCH(6K+/6E-), facilitating the VDW and ELE interactions between the charged residues on the second and third sheets. The single bilayer simulations have shown that the intra-sheet lysine and glutamic acid residues in CATCH(6K+/6E-) have stronger interactions than the intra-sheet lysine and aspartic acid residues in CATCH(6K+/6D-), -125.9 vs. -85.8 kcal/mol, respectively. For CATCH(6K+/6E-) there is not as large of an incentive to interact with sidechains on the opposing bilayer face as for CATCH 6K+/6D-).

**Table 2:** Summary of MMGBSA and LIE analysis for CATCH two stacked bilayer structures. MMGBSA values for VDW and ELE energies are calculated by considering the interactions between the top bilayer and the bottom bilayer. LIE values for VDW energies are calculated by considering only the sidechain-sidechain interactions between the charged residues on the top bilayer and the bottom bilayer. Values listed are averaged over three independent simulations.

System	VDW (kcal/mol)	ELE (kcal/mol)	VDW (LIE) (kcal/mol)
CATCH(6K+/6E-)	-67.0 ± 3.5	-1848.3 ± 471.2	-20.7 ± 4.4
CATCH(6K+/6D-)	-151.4 ± 24.3	-2355.1 ± 600.2	-49.8 ± 8.5

The difference in bilayer structure and face-to-face interactions between CATCH(6K+/6E-) and CATCH(6K+/6D-) provides a possible explanation for the difference in their experimentally-observed nanofiber structures. Cryogenic EM and TEM images show that CATCH(6K+/6E-) forms randomly entangled and tortuous nanofibers with short persistence lengths, whereas CATCH(6K+/6D-) forms aligned bundles of nanofibers with long persistence lengths that tend to appear as multi-layer stacks (Fig 2). The single bilayer atomistic simulations predict that CATCH(6K+/6E-) tends to form more twisted β-sheets than CATCH(6K+/6D-). As the β-sheets stack together, they must either untwist or twist together, both of which incur some energy cost [39,40]. However, for sheets with weak face-to-face attraction, i.e. CATCH(6K+/6E-), twisting comes at a lower cost. Generally, as fibril twisting decreases, the likelihood of β-sheet stacking and fibril thickness growth increases [40]. For CATCH(6K+/6E-), the twisted bilayer structure and weak face-to-face interactions are consistent with the formation of thin randomly entangled nanofibers which are not favored to stack or align. In contrast, for CATCH(6K+/6D-), the combination of a flat bilayer structure and strong face-to-face interactions between charged residues are consistent with the formation of multi-layer fibril bundles. The combination of strong intra-sheet interactions and weak face-to-face interactions leads to a twisted structure for CATCH(6K+/6E-), while the combination of weak intra-sheet interactions and strong face-to-face interactions lead to a flat structure for CATCH(6K+/6D-). This phenomenon has also been observed in atomistic MD simulations of short self-assembling peptides [39].

### Quantification of CATCH bilayer stacking

Three independent explicit-solvent atomistic MD simulations were carried out for a CATCH(6K+/6E-) mixture and for a CATCH(6K+/6D-) mixture, with each arranged in the two separated bilayer configuration. The two separated bilayer configurations are essentially the aforementioned two stacked bilayer configurations with an additional 10Å of space between the bilayers, for a total spacing of ~13Å between the edges of each bilayer. The additional spacing allowed the bilayers to have more choice in their stacking arrangement.

CATCH(6K+/6D-) had a greater number of contacts between charged residues on the 2nd and 3rd layer than CATCH(6K+/6E-), suggesting that CATCH(6K+/6D-) fibrils are more likely to be well-aligned than CATCH(6K+/6E-). CATCH(6K+/6D-) had 25,185 contacts between charged residues and an average twist angle of -2.2° between neighboring strands. CATCH(6K+/6E-) had 17,652 contacts between charged residues and an average twist angle of -2.8° between neighboring strands. Overall, our simulations suggest that the flatter bilayer observed for CATCH(6K+/6D-) leads to well-aligned fibrils.

### In-silico assessment of CATCH co-assembly pathway and co-assembly kinetics

**Fig 6:**
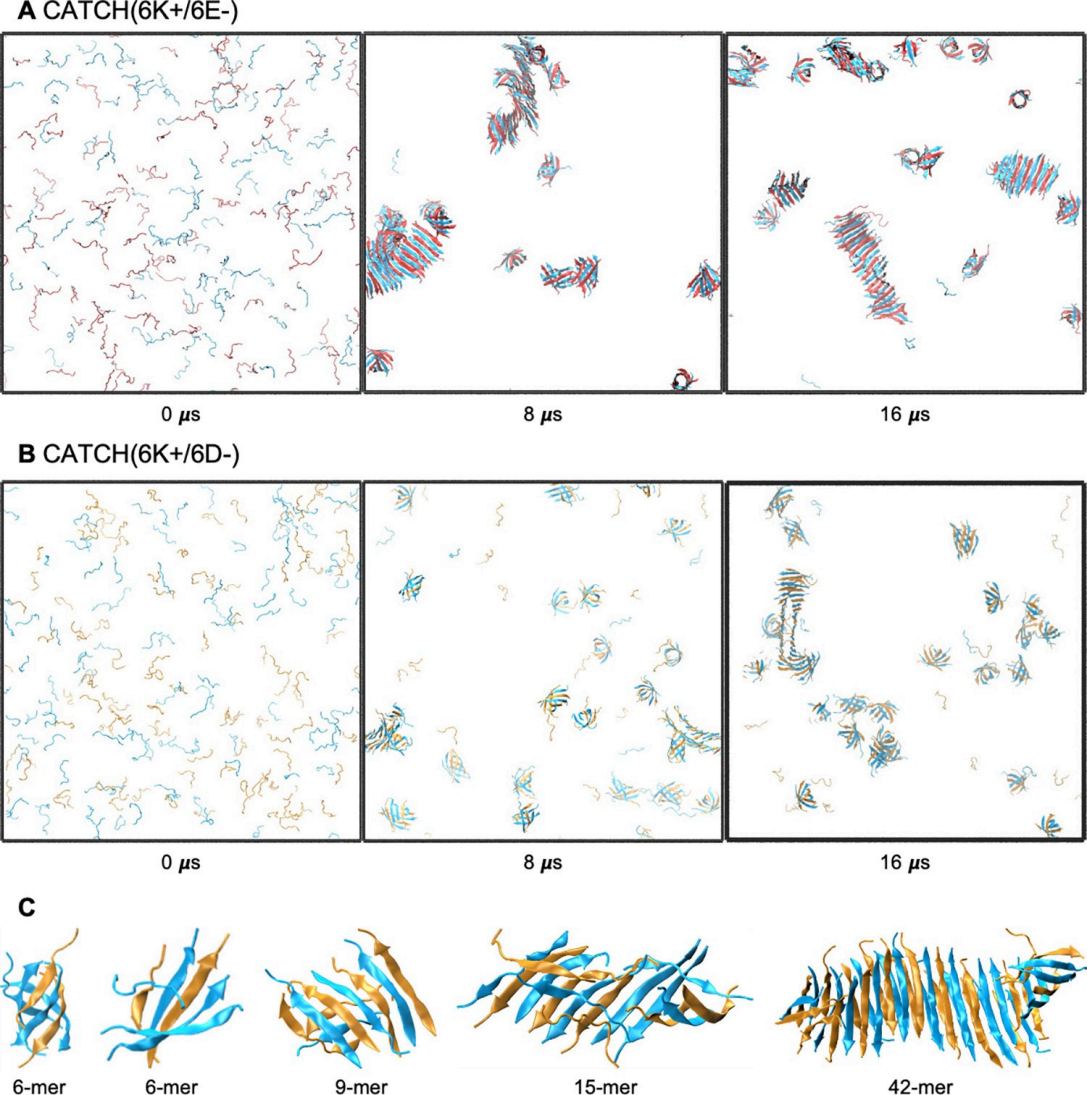
DMD Snapshots of **(A)** CATCH(6K+/6E-) and **(B)** CATCH(6K+/6D-) over the course of a 16 μs DMD simulation. Cationic peptides containing lysine are represented in teal. Anionic peptides containing aspartic acid are represented in orange, while anionic peptides containing glutamic acid are represented in red. **(C)** Chronological snapshots of oligomer growth, conformation change, and elongation of a β-barrel in the CATCH(6K+/6D-) simulation.

DMD/PRIME20 simulations of CATCH systems were carried out to determine their co-assembly pathway and co-assembly kinetics. Three independent systems of equimolar mixtures of CATCH(6K+/6E-) and of CATCH(6K+/6D-) containing 200 peptides, starting from random-coil configurations, were simulated for 500 billion collisions (~16 μs) at T* = 0.18 (296 K). Simulation snapshots at 0, 8, and 16 μs were taken to examine their assembly pathways (Fig 6A and 6B). At t = 0 μs, the peptides in the CATCH(6K+/6E-) and CATCH(6K+/6D-) systems are in random-coil conformations and are randomly arranged in the box. For CATCH(6K+/6E-), at t = 8 μs, nearly all the peptides have assembled into either a β-sheet structure or an ordered oligomer. At 16 μs, CATCH(6K+/6E-) has fully assembled into β-sheet structures and off-pathway β-barrels. For CATCH(6K+/6D-) at t = 8 μs, we see formation of a single β-sheet, multiple oligomers, and many free peptides still remaining. At t = 16 μs, we observed elongation of the previously-mentioned β-sheet, however there are still free peptides remaining. ThT assays have demonstrated that CATCH(6K/6E) assembles faster than CATCH(6K/6D) [6]. Given the timescale (~16 μs) of our simulations and the difference in assembly kinetics between CATCH(6K+/6E-) and CATCH(6K+/6D-), it is not unexpected to observe some “free” peptides in the CATCH(6K+/6D-) system. For both CATCH systems we observe β-sheet growth through monomer addition or through the interaction of two small-ordered structures.

**Fig 7:**
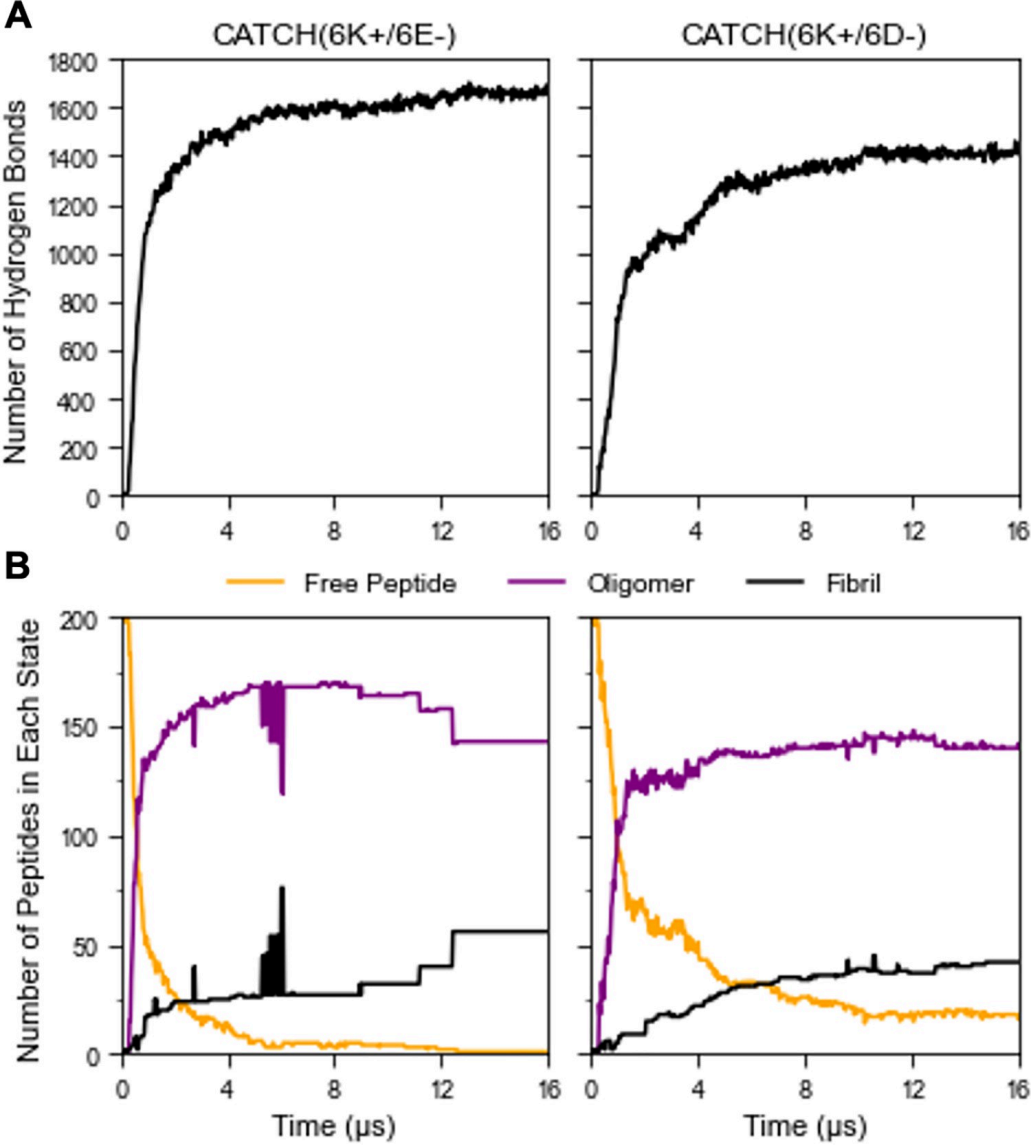
**(A)** Quantitative assessment of hydrogen bond formation over DMD simulation. (**B)** Analysis of free peptide depletion (orange), oligomerization (purple), and fibrillization (black).

Comparison of the hydrogen bond formation rates between CATCH(6K+/6E-) and CATCH(6K+/6D-) suggests that CATCH(6K+/6E-) fibrillizes at a faster rate than CATCH(6K+/6D-) (Fig 7A). The difference in assembly rates can be attributed to the difference in the anionic residue types. ThT analyses showed that CATCH(6K+/6E-) and CATCH(6K+/6D-) are both capable of assembling into β-sheet structures, however, CATCH(6K+/6E-) assembles at a significantly faster rate than CATCH(6K+/6D-) [6]. In experiments, this is likely related to the greater interaction strength between (K+/E-) pair than the (K+/D-) pair [35,36]. Due to its longer length, the glutamic acid sidechain in CATCH(6K+/6E-) has a greater range of interaction than the aspartic acid sidechain in CATCH(6K+/6D-), increasing the likelihood of finding the complementary lysine residue and forming backbone hydrogen bonds soon afterwards. By analyzing the rates of cluster formation and growth in CATCH(6K+/6E-) and CATCH(6K+/6D-) over time, we observe that CATCH(6K+/6E-) has a higher rate of β-sheet assembly than CATCH(6K+/6D-), and a greater depletion rate of free peptides, in agreement with the hydrogen bond kinetics (Fig 7) and ThT analyses.

Independent implicit-solvent atomistic REMD simulations of a single peptide were carried out for CATCH(6K+), (6E-), and (6D-) to explore the conformational space of CATCH free peptides in solution. Each peptide started in an extended conformation and was simulated for 200 ns at temperatures ranging from 310 to 600K. The DSSP (Define Secondary Structure of Proteins) algorithm was used to determine the average secondary content of each residue for trajectories at 310K (Table 3). Here, the term helix refers to 3–10, alpha, and pi helices. For convenient comparison, the sum of the averages for each peptide are also provided. CATCH(6K+), (6E-), and (6D-) all had helix, bend, turn, and coil conformations. Helices were found in all CATCH peptides between residues 3 and 9. CATCH(6E-) had the greatest total amount of helical content compared to CATCH(6K+) and (6D-), 6.27 vs 4.42 and 5.24 respectively. Notably, CATCH(6K+) had the most bend conformations compared to CATCH(6E-) and (6D-); CATCH(6K+) had a total bend conformation of 1.25, while CATCH(6E-) and (6D-) had a total bend conformation of 0.43 and 0.62, respectively. CATCH(6K+) and (6D-) had more coil content than CATCH(6E-), 2.39 vs. 1.63, respectively.

**Table 3:** Summary of DSSP analysis for each CATCH single peptide REMD simulation. Average secondary content over all frames for each residue are reported. Helix is the sum of the averages for 3–10, alpha, and pi helices. The total value is the sum of the averages over each CATCH single peptide.

	Helix			Bend			Turn			Coil		
Residue	(6K+)	(6E-)	(6D-)	(6K+)	(6E-)	(6D-)	(6K+)	(6E-)	(6D-)	(6K+)	(6E-)	(6D-)
**1**	0.23	0.24	0.20	0.00	0.00	0.00	0.29	0.22	0.19	0.48	0.54	0.61
**2**	0.39	0.47	0.37	0.00	0.00	0.00	0.32	0.27	0.25	0.28	0.27	0.37
**3**	0.47	0.62	0.54	0.18	0.08	0.10	0.24	0.23	0.24	0.09	0.07	0.12
**4**	0.50	0.66	0.60	0.19	0.09	0.09	0.23	0.21	0.25	0.08	0.04	0.06
**5**	0.48	0.69	0.63	0.18	0.07	0.09	0.24	0.18	0.21	0.10	0.07	0.08
**6**	0.50	0.72	0.64	0.21	0.07	0.09	0.22	0.19	0.22	0.06	0.03	0.05
**7**	0.55	0.75	0.63	0.13	0.04	0.07	0.27	0.18	0.21	0.05	0.03	0.09
**8**	0.48	0.74	0.60	0.20	0.04	0.10	0.26	0.19	0.25	0.06	0.02	0.05
**9**	0.43	0.66	0.50	0.16	0.04	0.09	0.32	0.26	0.30	0.09	0.04	0.12
**10**	0.27	0.46	0.34	0.00	0.00	0.00	0.31	0.39	0.35	0.41	0.16	0.31
**11**	0.12	0.28	0.19	0.00	0.00	0.00	0.18	0.34	0.29	0.70	0.39	0.52
**Total**	**4.42**	**6.27**	**5.24**	**1.25**	**0.43**	**0.62**	**2.88**	**2.66**	**2.75**	**2.39**	**1.63**	**2.39**

The difference in discordant helix content between CATCH(K+), (6E-), and (6D-) observed in atomistic REMD simulations provides additional insight into experimental co-assembly kinetics. A discordant helix is defined to be a helical segment in a peptide strand that has a tendency to form β-strands [41,42]. Our predicted conformations for CATCH(6E-) and CATCH(6D-) are in agreement with previously-observed conformations for aspartic acid oligomer and glutamic acid oligomer in simulations performed by Hunkler et. al. Their simulations showed that glutamic acid (E) oligomers tend to form stable α-helical structures, while aspartic acid (D) oligomers are intrinsically disordered [43]. In addition, glutamic acid has been frequently found in protein sequences with a propensity for helical structures [34,44,45].

The amount of α-helix/-beta-strand-discordance in CATCH(6E-) may facilitate β-sheet co-assembly. Kallberg et al. found that multiple amyloidogenic peptides that are predicted to form β-strands contain α-helices, and that fibril formation is lost upon removal or mutation of the α-helix-forming segments [41]. In a study on amyloid β-protein (Aβ), Fezoui and Teplow found that a partially-folded helix-containing conformer is an intermediate in Aβ fibril assembly, and that helix stabilization may facilitate fibril formation [46]. However, they also found that if a helix is sufficiently stabilized, it can resist structural reorganization and inhibit fibril formation [46,47]. The greater proportion of discordant helix observed in CATCH(6E-) than in (6D-) may promote faster β-sheet assembly in CATCH(6K+/6E-) than in (6K+/6D-).

More recent studies of Aβ aggregation suggest that a β-hairpin intermediate promotes dimer formation through the intermolecular β-bridges [48–51]. CATCH peptides are too short to properly organize into a β-hairpin. However, the bend conformation observed in CATCH(6K+) may play a similar role to the β-hairpin conformation observed in Aβ by acting as a partially stable intermediate that facilitates peptide-peptide interactions.

### β-barrels observed in DMD simulations of CATCH peptides

DMD/PRIME20 simulations also reveal on-pathway and off-pathway oligomers, including β-barrels in CATCH peptide co-assembly. In both CATCH(6K+/6E-) and CATCH(6K+/6D-) systems, β-barrels and ordered oligomers formed in addition to β-sheet structures. A β-barrel is a β-sheet that wraps around to form a cylindrical structure, with the last and first β-strands connected by backbone hydrogen bonds. β-barrels ranging in size from 6 to 8 peptides (Fig 6A and 6B) formed relatively early in the simulations for both CATCH(6K+/6E-) and CATCH(6K+/6D-). Nearly all the β-barrels that formed remained in a β-barrel structure throughout the course of the simulation—stabilized by hydrogen bonds and hydrophobic interactions between phenylalanine residues. The β-barrels that were not sufficiently stabilized by their intramolecular interactions unraveled to form β-sheets.

DMD simulations capture β-barrel-to-β-sheet transitions in both CATCH systems. Fig 6C shows a 6-mer β-barrel intermediate in the CATCH(6K+/6D-) system that eventually seeds the final 42-mer β-sheet structure. The DMD snapshots show the CATCH (6K+/6D-) β-barrel shifting into an elliptical shape, flattening out, and opening at the ends. The β-sheet then grows through a combination of monomer addition and interactions with other small ordered-structures containing β-strands, ultimately forming the final 42-mer β-sheet structure. β-barrel intermediates have also been observed by Sun et. al in atomistic DMD simulations of hIAPP19–29 and its S20G mutant, hIAPP22–28, Aβ16–22, and the α-synuclein NACore. Each of these peptides ultimately self-assembled into cross-β aggregates [52]. β-barrel oligomers have garnered a lot of interest in the field of neurodegenerative diseases as a source of toxicity and a possible therapeutic target [53]. Understanding what factors contribute to the formation of β-barrel oligomers can aid in designing new therapeutics. Our results suggest that the combination of charge complementarity and alternation of hydrophilic and hydrophobic residues used to design CATCH peptides may also promote the formation of β-barrel structures, similar to those found in amyloidogenic peptides and in agreement with conclusions drawn by Shao et. al on previous CATCH simulations [12]. Although CATCH peptides produce on-pathway and off-pathway β-barrels, our research provides insight into designing stable β-barrels with potential applications such as single-molecule sensors or DNA sequencing [54,55].

## Conclusion

In conclusion, we investigated the charged residue-residue interactions and the assembly pathway for two CATCH(+/-) pairs: CATCH(6K+/6E-) and CATCH(6K+/6D-). Although glutamic acid (E) only differs from aspartic acid (D) by one methylene group, previous studies have shown that this small difference in composition can lead to significant changes in structure at fibril and bulk material scale [45,56]. Our *in silico* results demonstrate that sidechain type plays a significant role in peptide co-assembly, and in turn, may affect the resulting fibril structure and morphology.

The difference in the CATCH(6K+/6E-) and CATCH(6K+/6D-) bilayer structures and face-to-face interactions observed in simulations provides a possible explanation for the difference in their nanofiber structures. In experiments CATCH(6K+/6E-) forms randomly entangled nanofibers, while CATCH(6K+/6D-) forms multi-layer stacks of aligned fibrils. Atomistic molecular dynamics simulations of single bilayers predict that CATCH(6K+/6E-) adopts a twisted structure, while CATCH(6K+/6D-) adopts a relatively flat β-sheet structure. The inherent twist in the CATCH(6K+/6E-) bilayer is due to the chirality of amino acids and strong inter-strand sidechain-sidechain interactions. However, the CATCH(6K+/6D-) bilayer is restricted from twisting to accommodate the backbone hydrogen bonding and salt bridge interactions between neighboring charged groups. Atomistic molecular dynamics simulations of two bilayers stacked on top of one another show that CATCH(6K+/6E-) has weaker face-to-face interactions between the two bilayers than CATCH(6K+/6D-). These results are further corroborated with MD simulations of two separated bilayers, that show that CATCH(6K+/6E-) has fewer number of contacts between the two bilayers than CATCH(6K+/6D-). For CATCH(6K+/6E-), the twisted bilayer structure and weak face-to-face interactions lead to formation of thin randomly entangled nanofibers. While for CATCH(6K+/6D-), the combination of a flat bilayer structure and strong face-to-face interactions between charged residues leads to formation of multi-layer fibril bundles.

Discontinuous molecular dynamics simulations with the PRIME20 forcefield reveal the β-sheet co-assembly pathway for CATCH(6K+/6E-) and CATCH(6K+/6D-). DMD/PRIME20 simulation results show that CATCH(6K+/6E-) co-assembles into β-sheet structures at a faster rate than CATCH(6K+/6D-), further substantiating Thioflavin T results showing faster assembly kinetics for CATCH(6K+/6E-) than for CATCH(6K+/6D-) [6]. The discordant helix observed in atomistic single peptide REMD simulations for CATCH(6E-) provides an additional possible explanation for the fast assembly kinetics observed experimentally for CATCH(6K+/6E-). Previous studies of discordant helices in Aβ peptides have shown that the presence of a helical component can lead to faster self-assembly than in the absence of the helical component [46]. However, further experimental and computational investigation on CATCH(6K+/6E-) and (6K+/6D-) dimerization and energy barriers for coil-to-β-sheet transitions are required to better understand CATCH co-assembly kinetics. We acknowledge that while the implicit solvent DMD and REMD simulations allow for longer simulation timescales, implicit solvent simulations by nature neglect the effect of solvent environment and counter-ion environment. The work presented focuses on sidechain-sidechain interactions and bilayer geometry. A future investigation on the competition between a loss in conformational entropy and a gain in counter-ion and solvent release entropy is necessary to determine the thermodynamic pathway for CATCH coassembly.

In addition to β-sheet formation, we also observed β-barrel intermediates in both CATCH(6K+/6E-) and CATCH(6K+/6D-) DMD simulations, similar to those found in atomistic MD simulations of amyloidogenic peptides by Sun et. al. [52] In future work, we hope to extend our investigation and explore the free-energy surface of CATCH intermediates to gain a better understand of CATCH coassembly.

Understanding how sidechain composition relates to assembly kinetics and ultimately to mechanical properties expands the bioengineer’s toolkit for peptide design. CATCH(+/-) peptides are cationic and anionic variants of Q11[QQKFQFQFEQQ]. The alternating motif of hydrophobic and hydrophilic residues is a common theme in self-assembling peptides and is maintained in the CATCH system. CATCH peptides are similar to intrinsically disordered region (IDR) sequences in that both are typically made up of charged residues [57]. The charged residues hinder two CATCH peptides of the same charge from self-assembling. However, CATCH peptides contain the hydrophobic residues which IDR sequences generally lack; the hydrophobic residues form the hydrophobic core of CATCH bilayers, creating an ordered structure with potential for fibril growth. There is still a daunting number of sequence mutations within the CATCH system to explore—and with each mutation, its effect on fibril structure. We envision that through a combination of computational and experimental collaboration, we can design β-strand structures (whether it be β-barrels or β-sheets) with precise organization, and subsequently, biomaterials with uniform properties.

## Supporting information

S1 Fig Final snapshots of CATCH(6K+/6E-) and CATCH(6K+/6E-) separated bilayer simulations after 200 ns MD simulation. (A) Top row shows side views for three independent simulations of CATCH(6K+/6E-) separated bilayer simulations. (B) Bottom row shows side views for three independent simulations of CATCH(6K+/6E-) separated bilayer simulations.
(TIF)
