# Supplementary figures and images for: Programming co-assembled peptide nanofiber morphology via anionic amino acid type: Insights from molecular dynamics simulations
Source: PLoS Comput Biol. 2023 Dec 4;19(12):e1011685. doi: 10.1371/journal.pcbi.1011685 (PMC10729967; doi:10.1371/journal.pcbi.1011685)

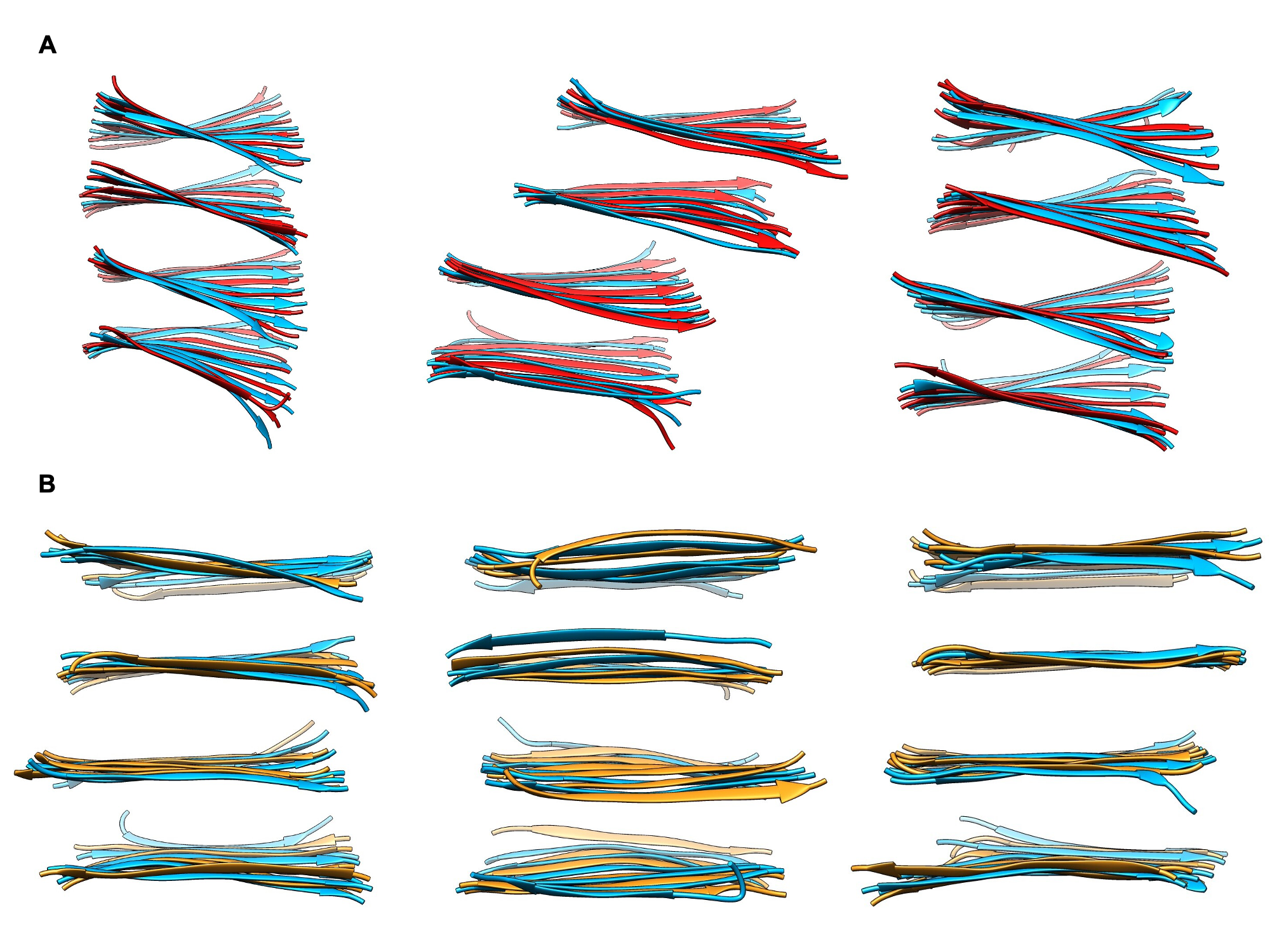

Supplement: S1 Fig — (A) Top row shows side views for three independent simulations of CATCH(6K+/6E-) separated bilayer simulations. (B) Bottom row shows side views for three independent simulations of CATCH(6K+/6E-) separated bilayer simulations. (TIF) [file pcbi.1011685.s001.tif]
